# A neutral homoaromatic heavy allene as a platform for selective conversion to a germylene-coordinated digermavinylidene

**DOI:** 10.1039/d5sc07177a

**Published:** 2025-10-20

**Authors:** Daichi Uchida, Hiroko Yamada, Yoshiyuki Mizuhata

**Affiliations:** a Institute for Chemical Research, Kyoto University Gokasho Uji Kyoto 611-0011 Japan mizu@boc.kuicr.kyoto-u.ac.jp

## Abstract

We report the synthesis of the first neutral homoaromatic heavy allene, stabilized by a methylene-bridged four-membered framework. Single-crystal X-ray diffraction and DFT calculations reveal a cyclic three-center–two-electron (3c–2e) π interaction, a short bridgehead distance, a delocalized HOMO, and a strongly negative NICS(−1) value (−16.9 ppm), collectively establishing pronounced homoaromaticity. Coordination of 4-dimethylaminopyridine (DMAP) induces a clean and selective transformation into a germylene-coordinated digermavinylidene, without substituent migration. This reactivity originates from LUMO localization at the bridgehead Ge atom supported by Wiberg bond indices and NBO analyses. Our findings highlight neutral homoaromaticity as a structural platform for programmable bonding interconversion in heavy main-group π systems.

## Abstract 2

The first neutral homoaromatic heavy allene enabling selective, migration-free conversion into a germylene-coordinated digermavinylidene.

## Graphical abstract

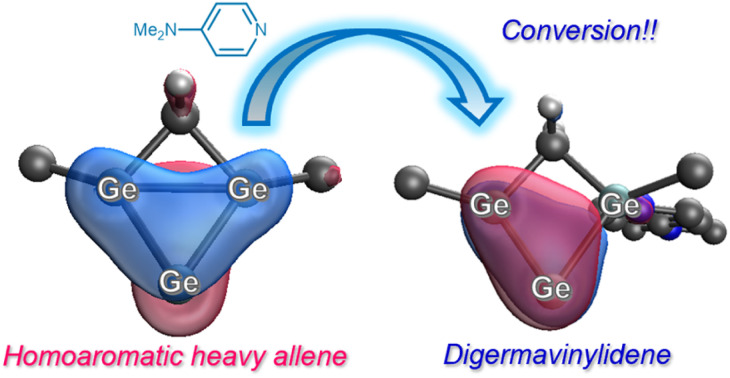

## Introduction

Allenes (R_2_C=C=CR_2_) are a fundamental class of π-systems defined by their linear C=C=C core and orthogonal double bonds. Carbon-based allenes are well understood and have found broad applications in synthesis and catalysis.^1–4^ By contrast, their heavier congeners (R_2_E=E=ER_2_; E = Group 14 elements beyond carbon) display drastically different geometries and electronic structures, reflecting the intrinsically poor pπ–pπ overlap and limited hybridization of heavy atoms. As a result, these compounds—often referred to as “heavy allenes” to distinguish them from their carbon analogues—deviate from linearity and are generally classified into three bonding types: bent allenes (Type I), ylidene forms (Type II), and ylidone structures (Type III). In reality, many heavy allenes adopt intermediate bonding descriptions, lying on a continuum between these archetypes. These variations highlight both the bonding preferences of heavy elements and their unconventional reactivity, making heavy allenes an attractive arena for main-group chemistry.

**Fig. 1:**
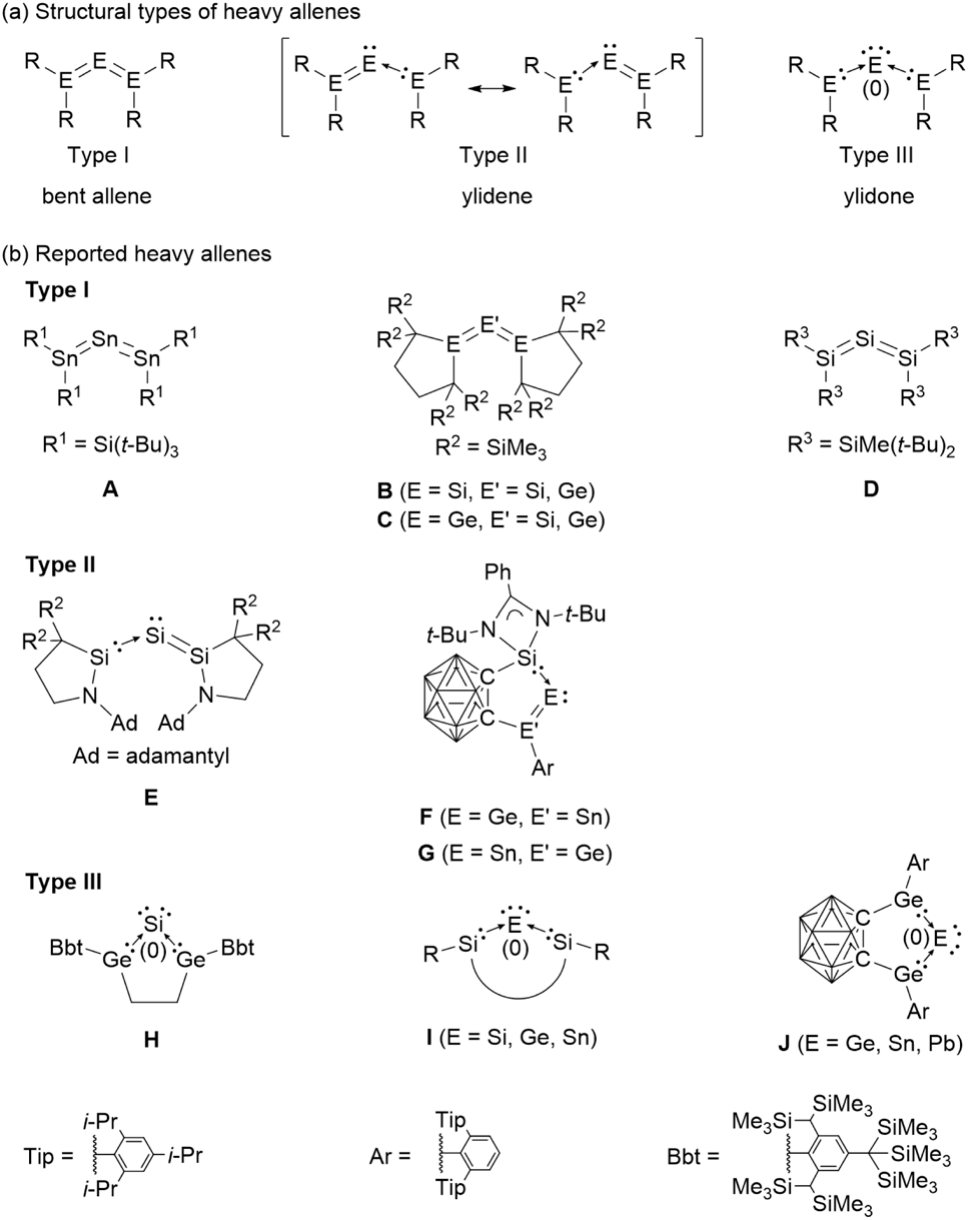
(a) Three structural types of heavy allenes. (b) Reported heavy allenes.

The first isolable heavy allene, a tristannaallene reported by Wiberg, was shown to adopt a bent structure (Fig. 1b, A).^5^^119^Sn NMR revealed low-valent character at the central tin atom, consistent with an alternative description as a stannylene-coordinated distannavinylidene (Type II). The syntheses of trisilaallene, trigermaallene, 1,3-digermasilaallene, and 2-germadisilaallene further established the field and demonstrated bent frameworks (Type I) as a common motif (Fig. 1b, B–D).^6–9^ More recently, ylidene-type heavy allenes (Type II) have been reported, and these studies revealed that the incorporation of donor substituents enabled modulation of the predominant resonance structures of heavy allenes (Fig. 1b, E–G).^10,11^

**Fig. 2:**
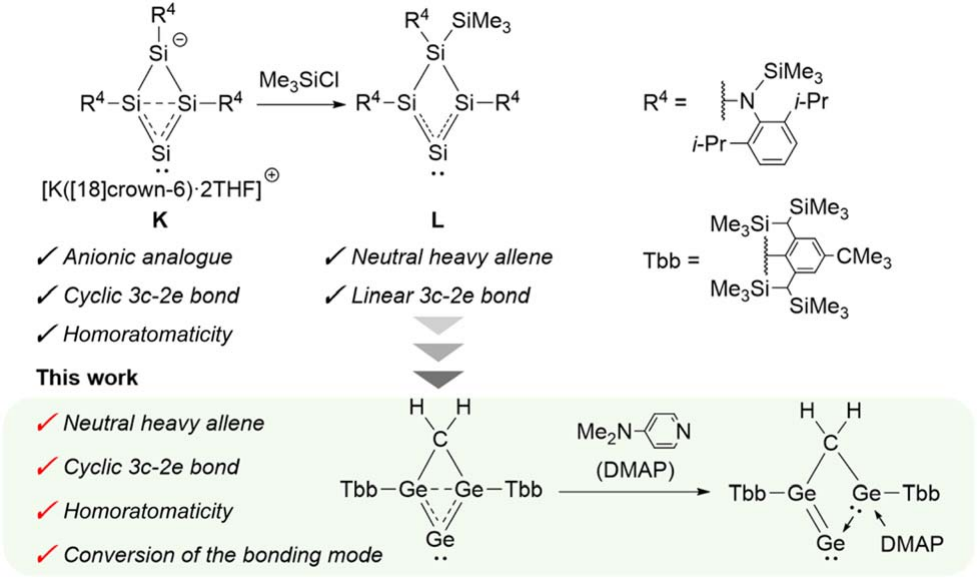
This work: design of a neutral homoaromatic allene and its conversion, building on insights from previous studies.^24^

In parallel, ylidone-type heavy allenes (Type III) have attracted growing attention because of their role in single-atom transfer reactions. Cyclic constraints have proven particularly powerful in enhancing the zero-valent character of the central atom, thereby favoring ylidone bonding. For example, the first cyclic heavy allene (H) displayed Si(0) character in ^29^Si NMR spectroscopy.^12^ A variety of cyclic heavy allenes with strong zero-valent contributions have since been prepared (Fig. 1b, I).^13–21^ A unifying feature is that donor coordination at the 1,3-positions amplifies the zero-valent nature of the central atom. More recently, cyclic frameworks incorporating carborane units (Fig. 1b, J) have been introduced, enabling single-atom transfer processes.^22,23^ In another striking example, an anionic analogue of a four-membered heavy allene has been reported, which reacts with trimethylsilyl chloride to afford the corresponding neutral heavy allene (Fig. 2, K and L).^24^ Interestingly, the anionic analogue K exhibits a cyclic three-center–two-electron (3c–2e) π interaction and thus clear homoaromatic character, whereas the neutral analogue L is dominated by a linear (allyl-type) 3c–2e delocalization and lacks genuine cyclic homoaromaticity. Collectively, these advances demonstrate that both cyclic frameworks and charge provide a versatile handle for tuning the structure and reactivity of heavy allenes. The extension of the concept of aromaticity to heavier main-group elements offers a general framework for designing novel reactive π-systems.

Motivated by these insights, our interest turned to a neutral homoaromatic allene, and we sought to design a new type of heavy allene featuring a methylene-bridged four-membered ring. A methylene group, being the most compact carbon linker, was expected to enforce close approach of the bridgehead atoms. Such enforced proximity could favor the formation of a cyclic 3c–2e bond across the heavy allene unit, introducing the possibility of homoaromatic stabilization.

Here, we report the synthesis of a structurally unique heavy allene of this type (Fig. 2). The compound displays clear homoaromaticity arising from a cyclic 3c–2e bond enforced by the methylene bridge, as supported by crystallographic and computational analysis. Coordination of 4-dimethylaminopyridine (DMAP) induces a selective transformation into a germylene-coordinated digermavinylidene. This study thus establishes a new structural paradigm for heavy allenes and provides a rare example of controllable bonding transformation in main-group π-systems.

## Results and discussion

**Scheme 1:**
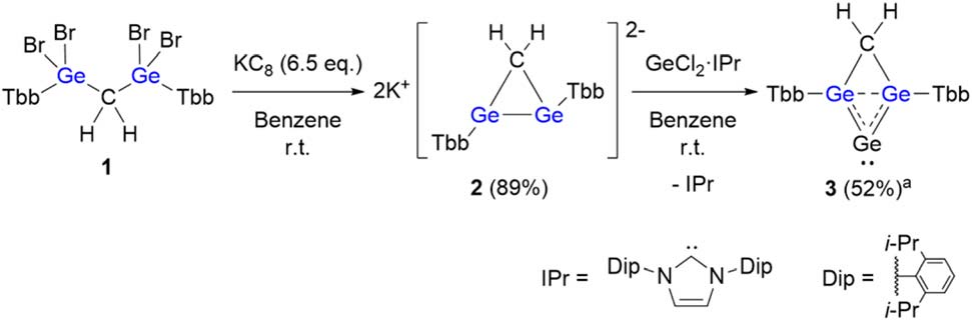
Synthesis of neutral homoaromatic allene 3. ^*a*^NMR yield.

**Fig. 3:**
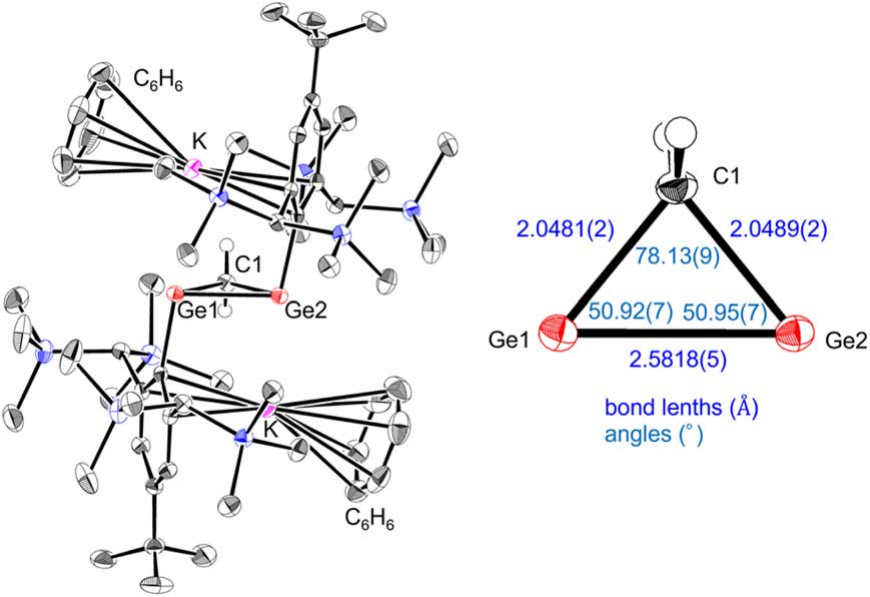
Molecular structure of 2·2C_6_H_6_ (thermal ellipsoid plots set at 50% probability). The hydrogen atoms except for CH_2_ are omitted for clarity.

To access the target framework, we employed a dianionic digermirane as a key precursor. Treatment of 1,1,3,3-tetrabromo-1,3-digermane (1)^25^ with 6.5 equivalents of KC_8_ afforded the dipotassium digermiran-1,2-diide (2) (Scheme 1). After removal of graphite and KBr by filtration, slow evaporation of the benzene solution at room temperature gave crystalline 2. Its structure was elucidated by NMR spectroscopy and single-crystal X-ray diffraction (Fig. 3, S1 and S2). The preparation of cyclopropane dianions remains a long-standing challenge in direct functionalization chemistry,^26,27^ and heavy-element analogues are exceedingly rare. Indeed, Lee and Sekiguchi reported a transient trisila analogue generated by two-electron reduction of a cyclotrisilene at cryogenic temperatures, which could not be isolated under ambient conditions.^28^ In contrast, 2 represents the first crystalline heavy cyclopropane dianion that is stable at room temperature.

The solid-state structure of 2 (Fig. 3) reveals crystallization as a contact ion pair, with potassium cations sandwiched between the solvated benzene molecule and the aryl substituents. The [Ge_2_C] core forms a nearly symmetric three-membered ring with equivalent Ge–C bond lengths [Ge1–C1: 2.0489(2) Å; Ge2–C1: 2.0481(2) Å]. These bonds are elongated relative to standard Ge–C single bonds (1.95–2.00 Å),^29^ consistent with repulsion from the high electron density on the anionic Ge centers. The Ge–Ge distance [2.5819(5) Å] is considerably longer than typical Ge–Ge single bonds (*ca.* 2.48 Å)^30^ and closely matches that in the long-bond isomer of 1,3-digermabicyclo[1.1.0]butane [2.5827(3) Å].^31^ Both Ge atoms adopt a distinct *trans*-pyramidal geometry [Ge1–Ge2–C(Tbb) 98.95(6)°, Ge2–Ge1–C(Tbb) 101.48(6)°], supporting localization of the negative charges at the germanium centers.

**Fig. 4:**
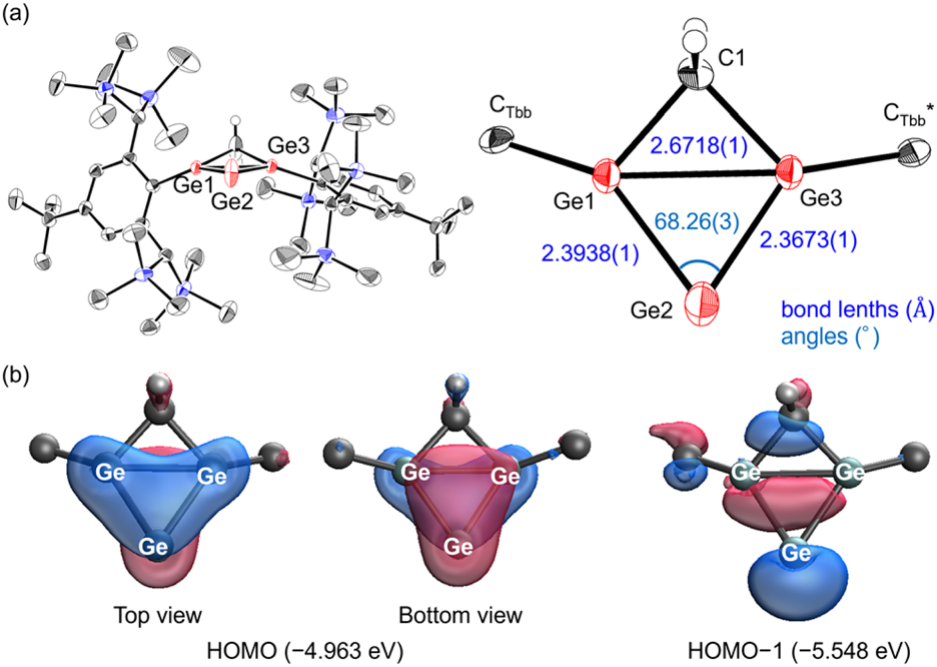
(a) Molecular structure of 3 (thermal ellipsoid plots set at 50% probability). The hydrogen atoms except for CH_2_ are omitted for clarity. (b) Molecular orbitals (isovalue: 0.05) of 3.

The reaction of 2 with GeCl_2_·IPr (IPr = 1,3-bis(2,6-diisopropylphenyl)imidazol-2-ylidene) proceeded rapidly to afford a red solid identified as homoaromatic heavy allene 3. The structure of 3 was confirmed by NMR spectroscopy and single-crystal X-ray diffraction (Fig. 4a, S4 and S6). Owing to the similar crystallization behavior of 3 and free IPr, separation by recrystallization proved difficult, and repeated attempts led to mixtures of products.

The molecular structure of 3, determined by single-crystal X-ray diffraction (Fig. 4a), consists of a four-membered [Ge_3_C] core with a *cis* arrangement of substituents. The structure was analysed as exhibiting pseudo-symmetric disorder in the [Ge_3_C] ring core (Fig. S11). The bond lengths between the bridge and bridgehead germanium atoms [Ge1–Ge2: 2.3938(1) Å; Ge2–Ge3: 2.3673(1) Å] lie within the range reported for digermenes [2.213(2)–2.416(2) Å].^32–38^ In contrast, the transannular Ge1–Ge3 distance [2.6718(1) Å] is longer than typical Ge–Ge single bonds (*ca.* 2.48 Å)^30^ but shorter than that of Ge–Ge π single-bonded species [2.8714(11) Å].^39^ These metrics indicate a weak but significant transannular interaction between the bridgehead atoms. The internal angle [Ge1–Ge2–Ge3: 68.26(3)°] is narrower than in known trigermallenes [C: 122.61(6)°, H: 82.27(5)°] or the neutral four-membered heavy allene [L: 73.24(4)°],^24^ highlighting the enforced proximity induced by the methylene bridge. Furthermore, the angle is comparable to that of anionic analogue K [65.21(3)°], strongly supporting the presence of homoaromaticity in 3.

Although the Si–Si and Ge–Ge separations are similar in magnitude [2.682(1) Å for L*vs.* 2.6718(1) Å for 3], their bonding interpretations differ fundamentally. The Si–Si distance is markedly elongated relative to a normal Si–Si single bond (*ca.* 2.34 Å), whereas the Ge–Ge distance is only slightly longer than a standard Ge–Ge single bond (*ca.* 2.48 Å). Thus, L is best described as allyl-type delocalization without transannular bonding, while 3 exhibits a genuine, though weak, transannular Ge–Ge interaction.

DFT calculations at the B3LYP-D3/6-311G(2df,2p) level reproduced the experimentally determined geometry of 3 with good fidelity (Table S3) and clarified its bonding picture (Fig. 4b). The HOMO is an asymmetric three-center–two-electron (3c–2e) π orbital delocalized over Ge1–Ge3 *via* Ge2; the asymmetry arises from the slight outward tilt of the Ge1/Ge3 p orbitals enforced by the *cis* arrangement of substituents. The HOMO−1 is a lone pair localized on Ge2, consistent with a formal zero-valent character at the bridge position. These assignments are supported by Wiberg bond indices (WBI: Ge1–Ge2 = 1.28, Ge2–Ge3 = 1.24, and Ge1–Ge3 = 0.44), which indicate significant double-bond character to the bridge atom and a measurable transannular interaction between the bridgeheads. Natural bond orbital analysis further shows nearly balanced contributions of the three Ge centers to the 3c–2e orbital [Ge1: 33% (practically pure p-orbital), Ge2: 36% (practically pure p-orbital), and Ge3: 31% (practically pure p-orbital)].

The homoaromatic nature is quantified by NICS calculations: NICS(−1) = −16.9 ppm at a point 1 Å above the ring center on the substituent side, whereas NICS(+1) = −5.6 ppm at the opposite face. The strongly negative NICS(−1) value evidences a diatropic ring current, comparable to those of the homoaromatic silatrigermacyclobutenylium ion (−17 ppm)^40^ and the germanium-containing bishomocyclopropenylium ion (−11 ppm).^41^ These analogies demonstrate that 3 is a rare neutral homoaromatic heavy allene.

**Scheme 2:**
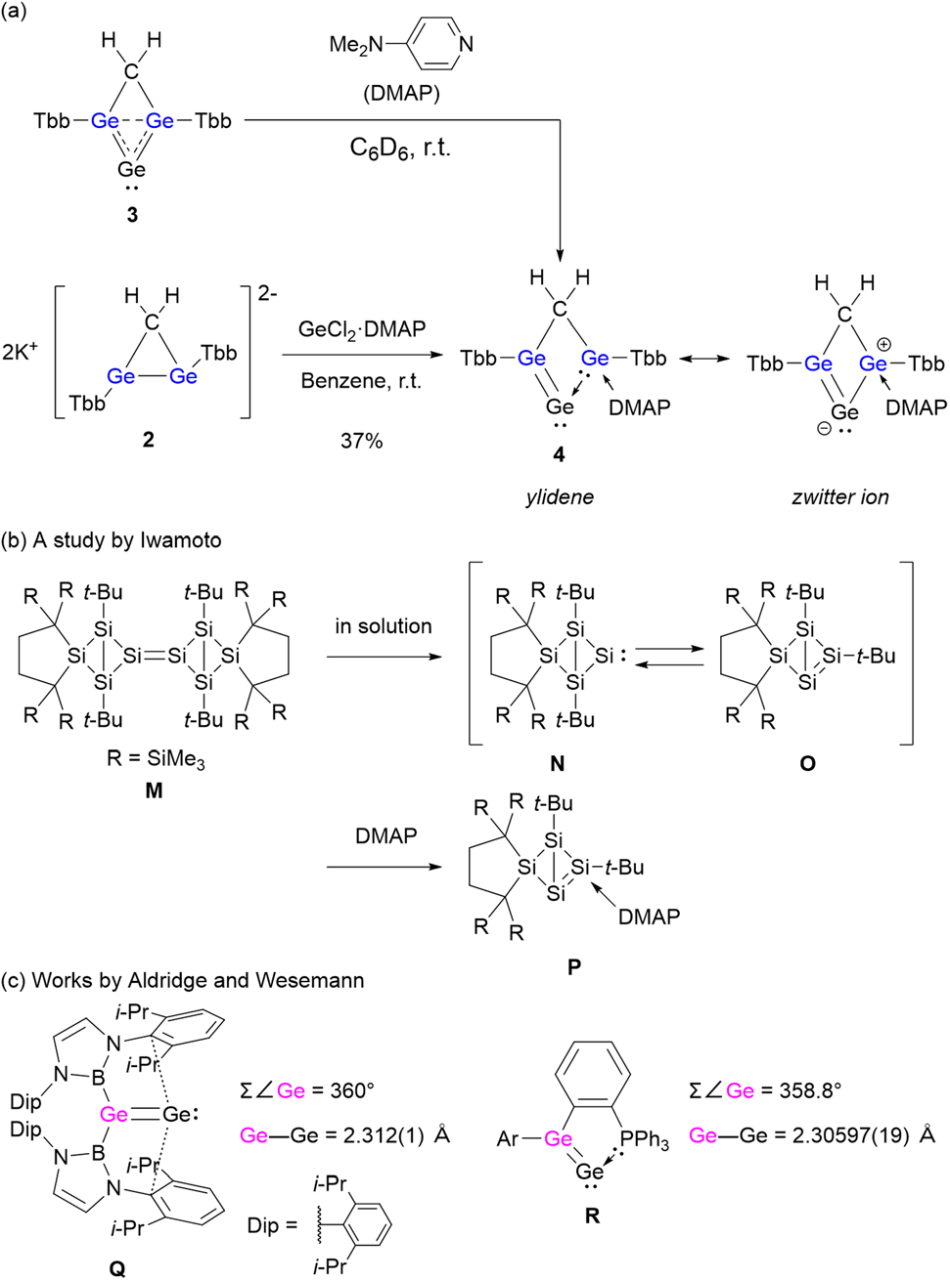
(a) Conversion of 3 into germylene-coordinated digermavinylidene 4. (b) Generation and isomerization of the silicon analogue of 3.^42^ (c) Reported digermavinylidenes.^43,44^

Upon treatment with 4-dimethylaminopyridine (DMAP), compound 3 was cleanly converted into digermavinylidene 4 (Scheme 2a). This transformation is particularly noteworthy when compared with the silicon analogue N,^42^ which is generated by dissociation of the dimer M (Scheme 2b). In that case, a *t*-Bu group migrates from the bridgehead to the bridge position to form isomer O, and only then does DMAP coordinate to yield P. In contrast, the germanium compound 3 remains monomeric and undergoes conversion to 4 without any substituent migration. This difference can be rationalized by the stabilization provided by the 3c–2e interaction in 3 and by the localization of the LUMO at the bridgehead Ge atom (Fig. S13). The transformation could also be observed in the ^1^H NMR spectrum of a mixture of 3 and DMAP, and compound 4 was successfully isolated by treatment of 3 with GeCl_2_·DMAP, affording a yellow solid in 37% yield. The absence of IPr coordination during the synthesis of 3 is attributed to steric hindrance caused by both the bulky Tbb substituents and the IPr ligand itself. While two Tbb groups of 4 are structurally asymmetric, they were observed as equivalent in the ^1^H and ^13^C{^1^H} NMR spectra. We interpret that this equivalence is due to a 1,3-shift mechanism of DMAP, and this process occurs faster than the NMR timescale (Fig. S7 and S8).

**Fig. 5:**
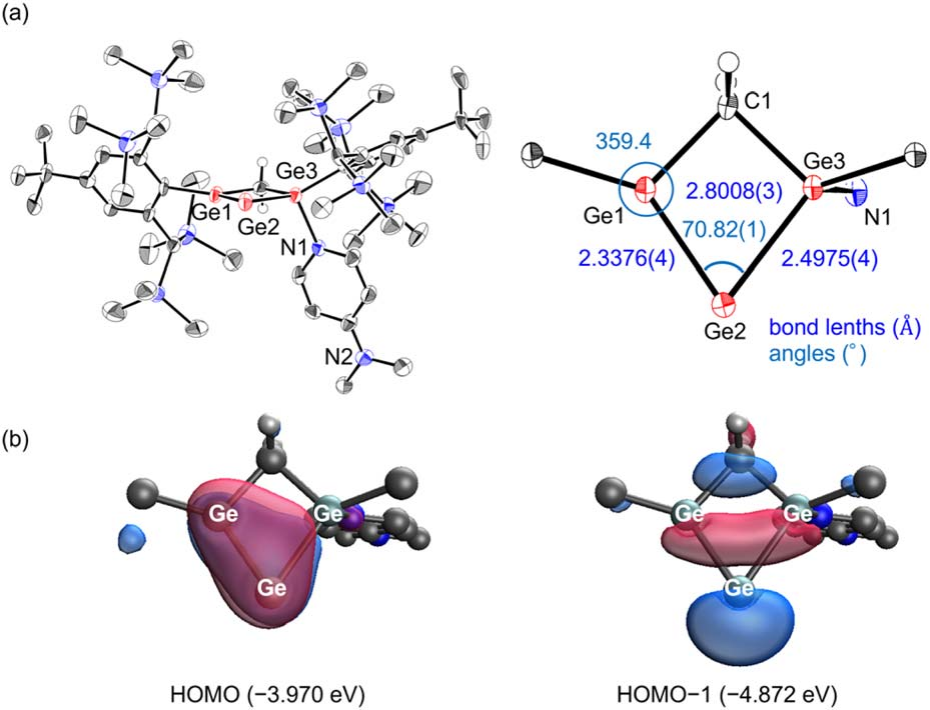
(a) Molecular structure of 4 (thermal ellipsoid plots set at 50% probability). The hydrogen atoms except for CH_2_ are omitted for clarity. (b) Molecular orbitals (isovalue: 0.05) of 4.

The molecular structure of 4, determined by single-crystal X-ray diffraction (Fig. 5a), exhibits a pronounced change in bonding relative to 3. The Ge1–Ge2 bond shortens to 2.3376(4) Å, while the Ge2–Ge3 bond elongates to 2.4975(4) Å and the transannular Ge1–Ge3 distance increases to 2.8008(3) Å. For comparison, the corresponding bond lengths in 3 are Ge1–Ge2: 2.3938(1) Å and Ge2–Ge3: 2.3673(1) Å. The shortened Ge1–Ge2 bond in 4 is consistent with values reported for digermavinylidenes [Q: 2.312(1) Å; R: 2.30597(19) Å].^43,44^ The internal angle [Ge1–Ge2–Ge3: 70.82(1)°] remains narrow for a heavy allene, though wider than that in 3. In addition, the sum of bond angles around Ge1 is 359.4°, essentially planar, which is comparable to the values reported for other digermavinylidenes [Q: 360.0°; R: 358.8°] (Scheme 2c).^43,44^ These structural parameters firmly establish that 4 adopts a germylene-coordinated digermavinylidene framework.

Computational analyses (DFT, NBO, WBI, and NPA) provide further insight into the bonding situation of 4 (Fig. 5b). The HOMO is a π orbital between Ge1 and Ge2, while the HOMO−1 corresponds to a lone pair on Ge2. Wiberg bond indices confirm a strong double-bond character between Ge1 and Ge2 (1.61) and a weaker interaction between Ge1 and Ge3 (0.27) compared to that of 3. Natural bond orbital analysis reveals that the Ge1–Ge2 σ bond consists of 61% contribution from Ge1 (sp^1.16^) and 39% from Ge2 (sp^7.88^d^0.06^). The lone pair at Ge2 is essentially pure s in character (sp^0.23^), while the π bond between Ge1 and Ge2 is distributed nearly evenly (52% on Ge1, 48% on Ge2) with almost pure p contributions on both atoms. Second-order perturbation analysis of 4 revealed that the Ge1–Ge2 π orbital significantly interacts with the π* orbitals on Ge3 (31.74 kcal mol^−1^). These results suggest that the vinylidene character is predominant in 4, with a slight contribution of a 3c–2e interaction. Natural population analysis (NPA) assigns charges of +0.65 (Ge1), −0.25 (Ge2), and +1.16 (Ge3), indicating pronounced zwitterionic polarization across the [Ge_3_C] framework. These computational results, together with the crystallographic data, firmly establish 4 as a germylene-coordinated digermavinylidene.

It is also worth noting that related transformations, in which Lewis base coordination induces conversion of heavy allenes into ylidone-type species, have been reported in a few recent studies.^10,23^ In this context, the present system provides a particularly well-defined example where a homoaromatic allene stabilized by a cyclic 3c–2e interaction is selectively disrupted by base coordination, establishing a new design principle for controllable bonding transformation in heavy-element π systems.

## Conclusions

In summary, we synthesized the first neutral homoaromatic heavy allene and demonstrated its clean and selective conversion into a germylene-coordinated digermavinylidene upon DMAP coordination, without substituent migration. Single-crystal X-ray diffraction, DFT, NBO, WBI, and NICS analyses collectively establish that compound 3 is stabilized by a cyclic three-center–two-electron π interaction, representing a rare example of a neutral homoaromatic species in heavy main-group chemistry. The contrasting bonding motifs of 3 and 4 highlight the structural programmability of heavy-element π systems: homoaromaticity enforced by a cyclic 3c–2e bond can be disrupted in a controlled manner and redirected into a ylidene-type bonding mode by Lewis base coordination. Looking ahead, this concept of programmable bonding interconversion can be extended to other Group 14 π frameworks and may guide the rational design of functional molecular materials and novel reactivity patterns.

## Author contributions

Y. M. conceived and supervised the project. D. U. performed the synthetic experiments and analysed the data. Y. M. and D. U. performed theoretical calculations. H. Y. and Y. M. participated in the discussions. D. U. and Y. M. cowrote the manuscript. H. Y. revised and polished the manuscript. All authors discussed the results and commented on the manuscript.

## Conflicts of interest

There are no conflicts to declare.

## Supplementary Material

SC-OLF-D5SC07177A-s001

SC-OLF-D5SC07177A-s002

SC-OLF-D5SC07177A-s003

## Data Availability

CCDC 2485173–2485175 contain the supplementary crystallographic data for this paper.^45*a*–*c*^ Supplementary information: the experimental data, analytical results, and additional supporting information related to this study, including details of experimental synthesis, the UV-vis spectra, NMR spectra and X-ray crystallographic data. See DOI: https://doi.org/10.1039/d5sc07177a.
