# Supplementary material for: A neutral homoaromatic heavy allene as a platform for selective conversion to a germylene-coordinated digermavinylidene
Source: Chem Sci. 2025 Oct 20;16(47):22597–602. doi: 10.1039/d5sc07177a (PMC12560044; doi:10.1039/d5sc07177a)
Supplement: SC-016-D5SC07177A-s002 [file SC-016-D5SC07177A-s002.pdf]

Supplementary Information (SI) for Chemical Science.
This journal is © The Royal Society of Chemistry 2025

Table of Contents

Experimental Procedures	S1
NMR Spectra of New Compounds	S3
UV/vis Spectra	S7
X-Ray Diffraction Studies	S8
Theoretical Calculations	S10
References	S12
Author Contributions	S12

Experimental Procedures

General information

All experiments were performed under an argon atmosphere in Yamato YGB1-CS glovebox. Anhydrous benzene-*d*$_6$ and dibromomethane were obtained by Freeze-Pump-Thaw followed by drying in molecular sieves 4A. Benzene was purified by The Ultimate Solvent System (Glass Contour Company). Compound **1** was prepared according to the reported procedures.[1] Potassium graphite (KC_8) was prepared by heating an 8:1 mixture of graphite and potassium.

^{1}H and ^{13}C NMR spectra were measured on a Bruker Ascend^{TM} 400 (^{1}H: 400 MHz, ^{13}C: 101 MHz). For the ^{1}H NMR spectra, signal arising from residual partially hydrogenated C_6D_5H (7.16 ppm) was used as the reference. The signal of C_6D_6 (128.06 ppm) was used for the ^{13}C NMR spectrum. The multiplicity of the signals in the ^{13}C NMR spectra was determined by DEPT techniques. UV/vis absorption spectra were recorded on a JASCO V-780 spectrophotometer in 0.1 cm quartz cells. All melting points were determined on a BÜCHI melting point M-565 and are uncorrected. Elemental analyses were carried out at the Microanalytical Laboratory (Institute for Chemical Research) of Kyoto University.

Synthesis of dipotassium digermiran-1,2-diide 2.

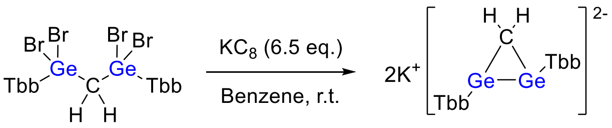

1 **2**

To a solution of $(TbbGeBr_2)_2CH_2$ (**3**) (99.5 mg, 0.0722 mmol) in benzene (5 mL) was added KC_8 (63.4 mg, 0.469 mmol) at room temperature, and stirred for 6 h. After filtration and removal of solvents in vacuo, the obtained mixture was purified by recrystallization from *n*-hexane at room temperature. Dipotassium digermirane-1,2-diide (**2**) (72.9 mg, 0.0641 mmol, 47%) was given as a red solid.

2; mp 134.8 °C (decomp.);δ ^{1}H NMR (400 MHz, C_6D_6, 298 K): δ −0.42 (s, 2H, CH_2), 0.40 (s, 36H, $SiMe_3$) 0.44 (br s, 36H, $SiMe_3$), 1.21 (s, 18H, *t*-Bu), 4.54 (br s, 4H, *o*-benzyl-*H*), 6.56 (s, 4H, Ar*H*); ^{13}C{^{1}H} NMR (101 MHz, C_6D_6, 298 K): δ 2.39 (q, Si(CH$_3$)$_3$), 26.9 (t, CH$_2$), 31.3 (d, benzyl-*C*), 31.6 (q, C(CH$_3$)$_3$), 33.8 (s, C(CH$_3$)$_3$), 120.4 (d, Ar*C*), 142.2 (s, Ar*C*), 151.0 (s, Ar*C*), 169.8 (s, Ar*C*); UV/vis spectrum (in benzene) λ_{max} (ε) 349.8 nm (7200), 507.4 nm (2954); Anal. calcd. for $C_{49}H_{100}Ge_2Si_8K_2$: C 51.74; H 8.86; Found: C 51.50; H 8.94.

Synthesis of homoaromatic heavy allene 3.

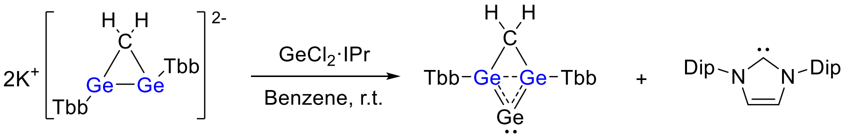

2 **3** IPr

To a solution of dipotassium digermiran-1,2-diide **2** (43.4 mg, 0.0336 mmol) in benzene (5 mL) was added $GeCl_2$·IPr (17.9 mg, 0.0336 mmol) at room temperature, and the solution was stirred for 30 min. After the solvent was removed in vacuo, the obtained mixture was recrystallized from *n*-hexane at room temperature, and red crystals suitable for single-crystal X-ray diffraction of **3** were obtained. Although repeated recrystallization led to the decomposition of **3**, making its isolation difficult, the reaction was found to produce 52% (**3**) and 97% (IPr) in NMR yield, using hexamethylbenzene as an internal standard.

3; ^{1}H NMR (400 MHz, C_6D_6, 298 K): δ 0.25 (s, 72H, Si(CH_3)$_3$), 1.32 (s, 18H, C(CH_3)$_3$), 2.94 (s, 4H, Tbb-*o*-benzyl-*H*), 4.71 (s, 2H, CH_2), 6.99 (s, 4H, Tbb-Ar*H*); ^{13}C{^{1}H} NMR (101 MHz, C_6D_6, 298 K): δ 0.89 (q, Si(CH$_3$)$_3$), 31.3 (q, C(CH$_3$)$_3$), 32.8 (d, benzyl-*C*), 34.7 (s, C(CH$_3$)$_3$), 77.3 (t, CH$_2$), 121.6 (d, Ar*C*), 148.0 (s, Ar*C*), 148.3 (s, Ar*C*), 152.7 (s, Ar*C*).

Synthesis of germylene-coordinated digermavinylidene 4.

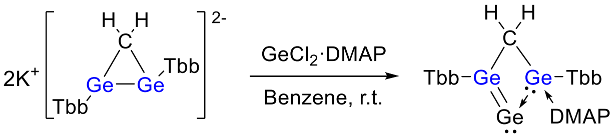

2 **4**

To a solution of dipotassium digermiran-1,2-diide **2** (36.0 mg, 0.0278 mmol) in benzene (5 mL) was added $GeCl_2$·IPr (7.4 mg, 0.028 mmol) at room temperature, and the solution was stirred for 30 min. After filtration and removal of solvents in vacuo, germylene-coordinated digermavinylidene **4** (12.8 mg, 0.0102 mmol, 37%) was given as a yellow solid.

4; mp 106.1 °C (decomp.); ^{1}H NMR (400 MHz, C_6D_6, 298 K) δ 0.32 (s, 72H, Si(CH_3)$_3$), 1.40 (s, 18H, C(CH_3)$_3$), 1.99 (s, 6H, N(CH_3)$_2$), 3.52 (br s, 4H, Tbb-*o*-benzyl-*H*), 3.87 (br s, 4H, CH_2), 6.04 (d, ^3J = 6.1 Hz, 2H, DMAP-Ar*H*), 7.0 (s, 4H, Ar*H*), 8.77 (d, ^3J = 6.1 Hz, 2H, DMAP-Ar*H*); ^{13}C{^{1}H} NMR (101 MHz, C_6D_6, 298 K): δ 1.39 (q, Si(CH$_3$)$_3$), 30.5 (d, benzyl-*C*), 31.5 (q, C(CH$_3$)$_3$), 34.6(s, C(CH$_3$)$_3$), 38.3 (q, N(CH$_3$)$_2$), 67.4 (t, CH$_2$), 106.8 (d, ArC) 122.2 (d, ArC), 145.6 (s, Ar*C*), 148.2 (d, ArC), 149.0 (s, Ar*C*), 150.6 (s, Ar*C*), 155.3 (s, Ar*C*); UV/vis spectrum (in benzene) λ_{max} (ε) 432.8 nm (374); Anal. calcd. for $C_{56}H_{110}Ge_3Si_8N_2$: C 53.63; H 8.80; N 2.23; Found: C 53.57; H 8.64; N 2.35.

NMR Spectra of New Compounds

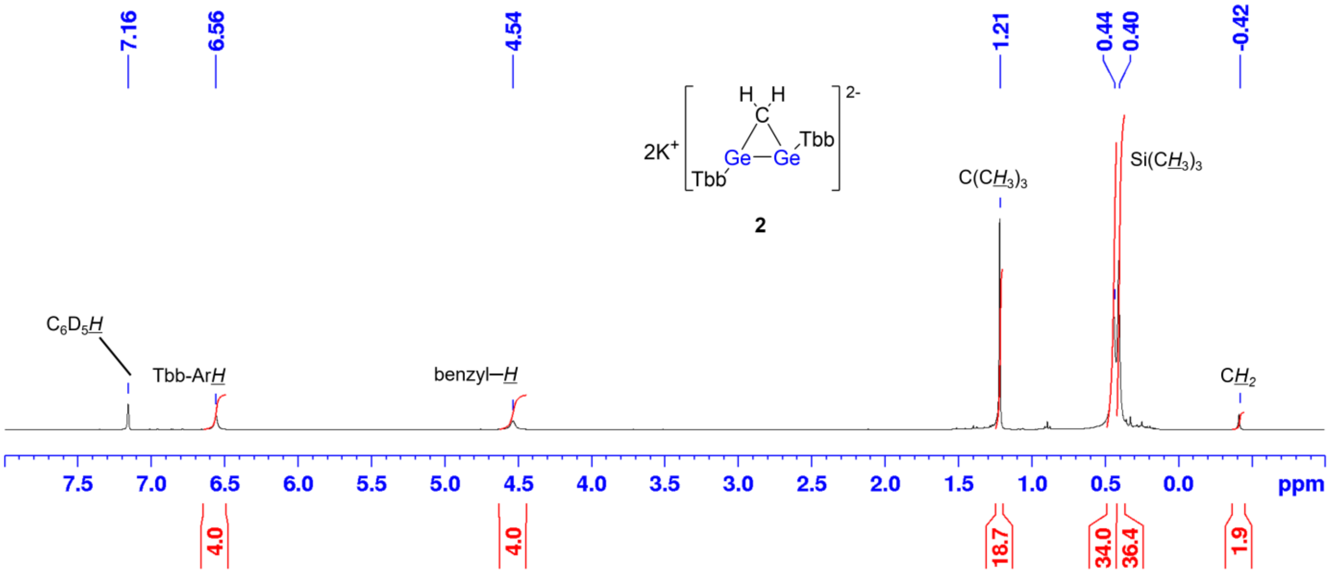

Figure S1. ^{1}H NMR spectrum of **2** (400 MHz, 298 K, C_6D_6).

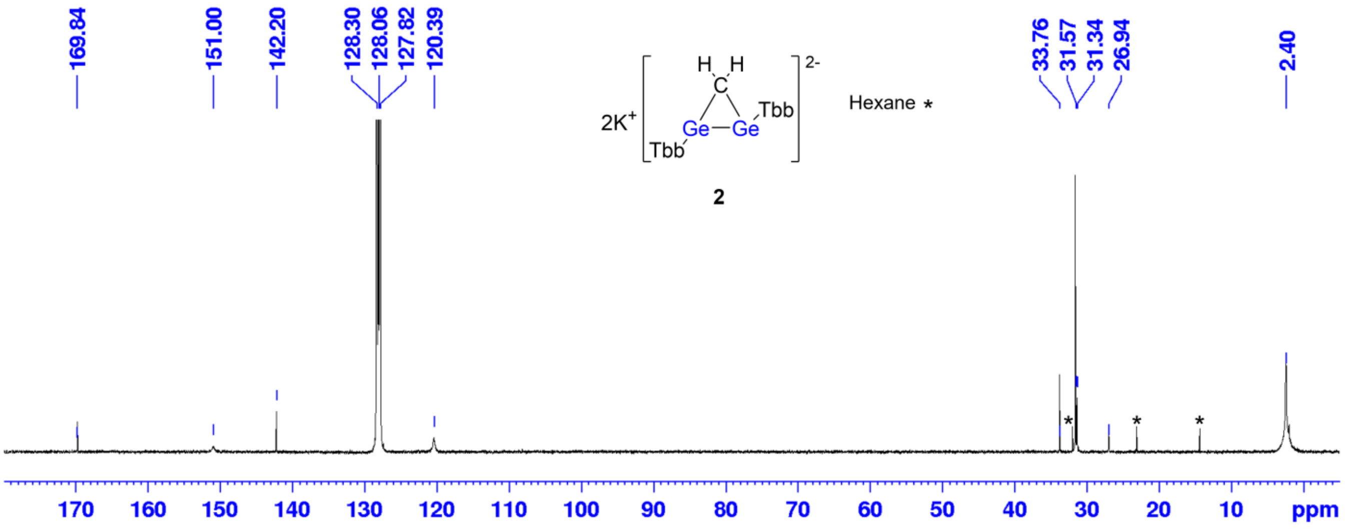

Figure S2. $^{13}C\{^{1}H\}$ NMR spectrum of **2** (101 MHz, 298 K, C_6D_6).

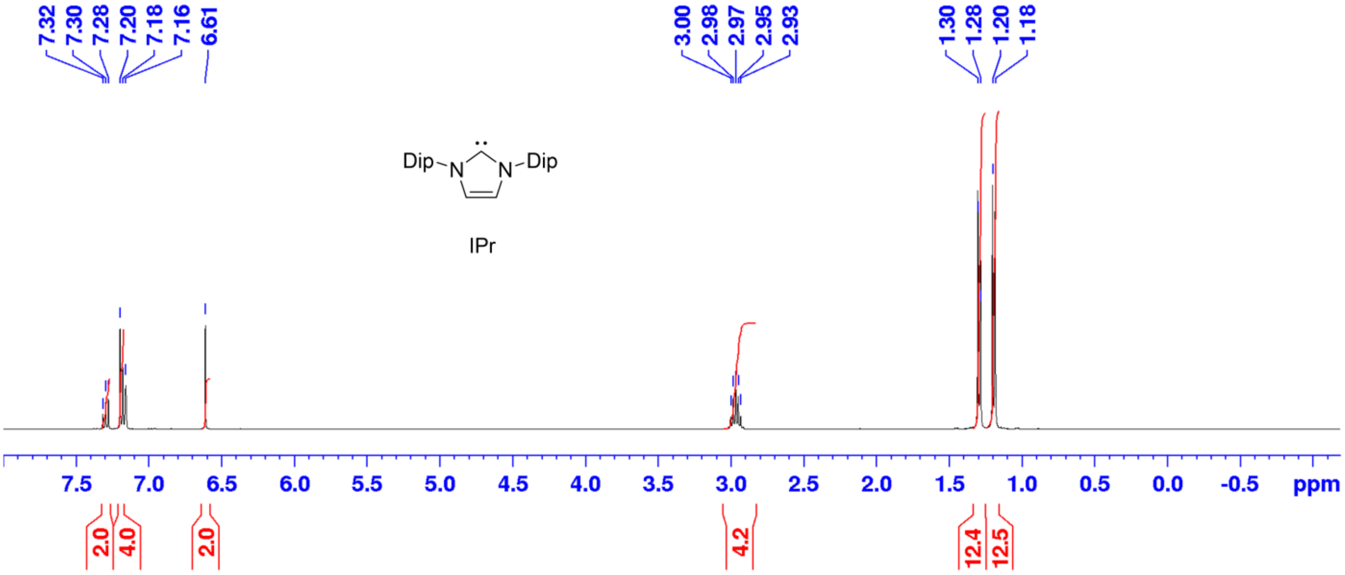

Figure S3. ${}^{1}H$ NMR spectrum of IPr (400 MHz, 298 K, C_6D_6).

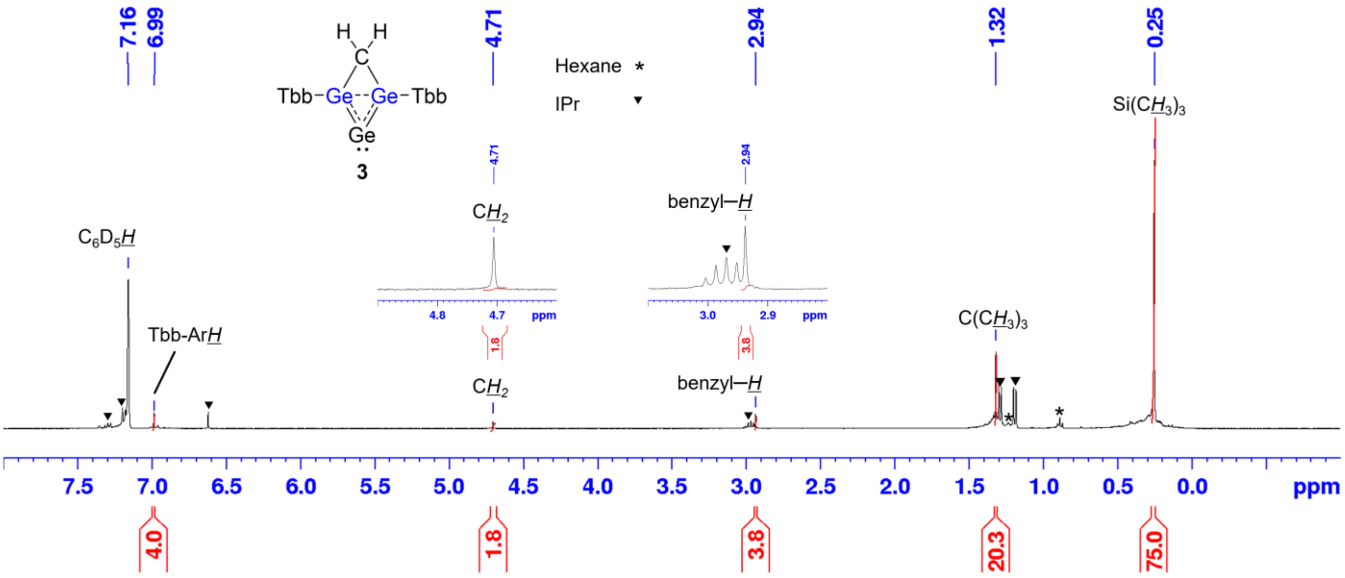

Figure S4. ${}^{1}H$ NMR spectrum of **3** (400 MHz, 298 K, C_6D_6).

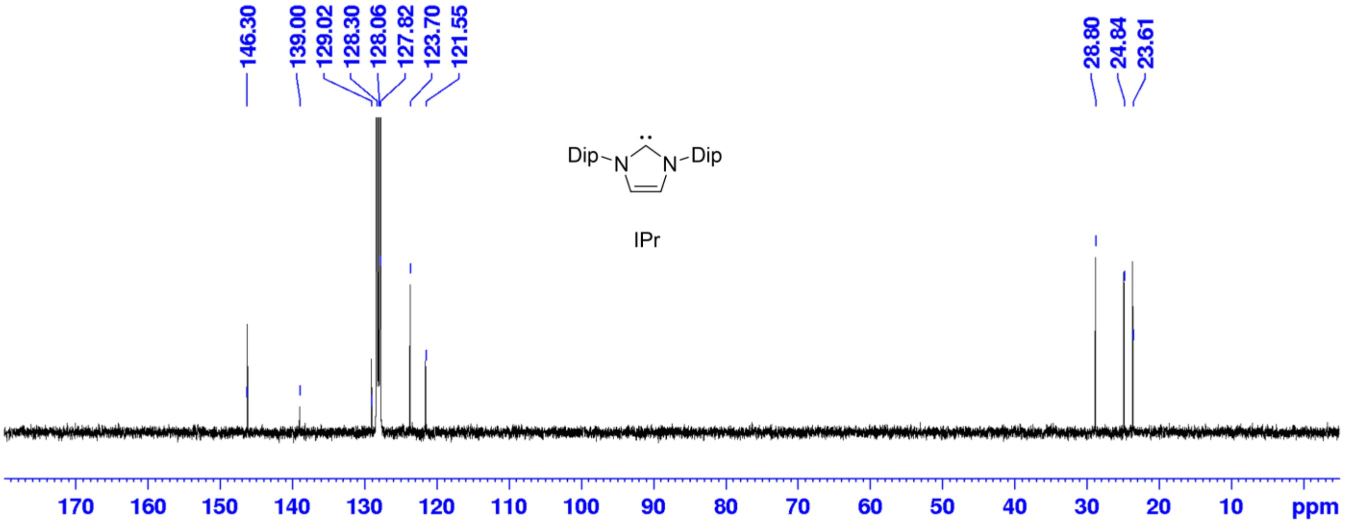

Figure S5. $^{13}C\{^{1}H\}$ NMR spectrum of IPr (101 MHz, 298 K, C_6D_6).

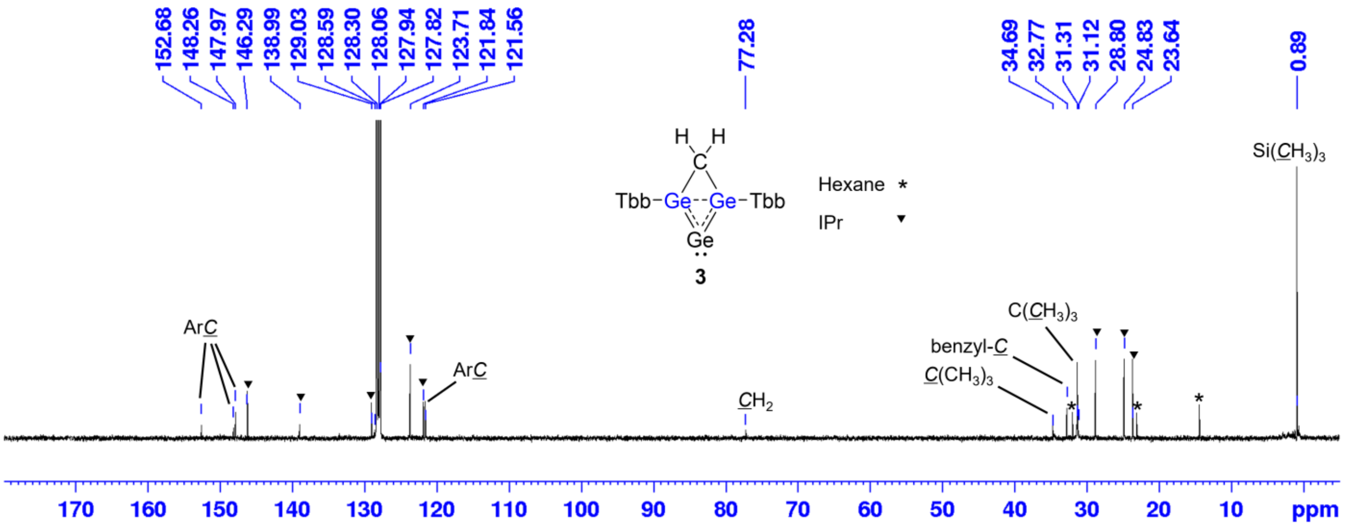

Figure S6. $^{13}C\{^{1}H\}$ NMR spectrum of **3** (101 MHz, 298 K, C_6D_6).

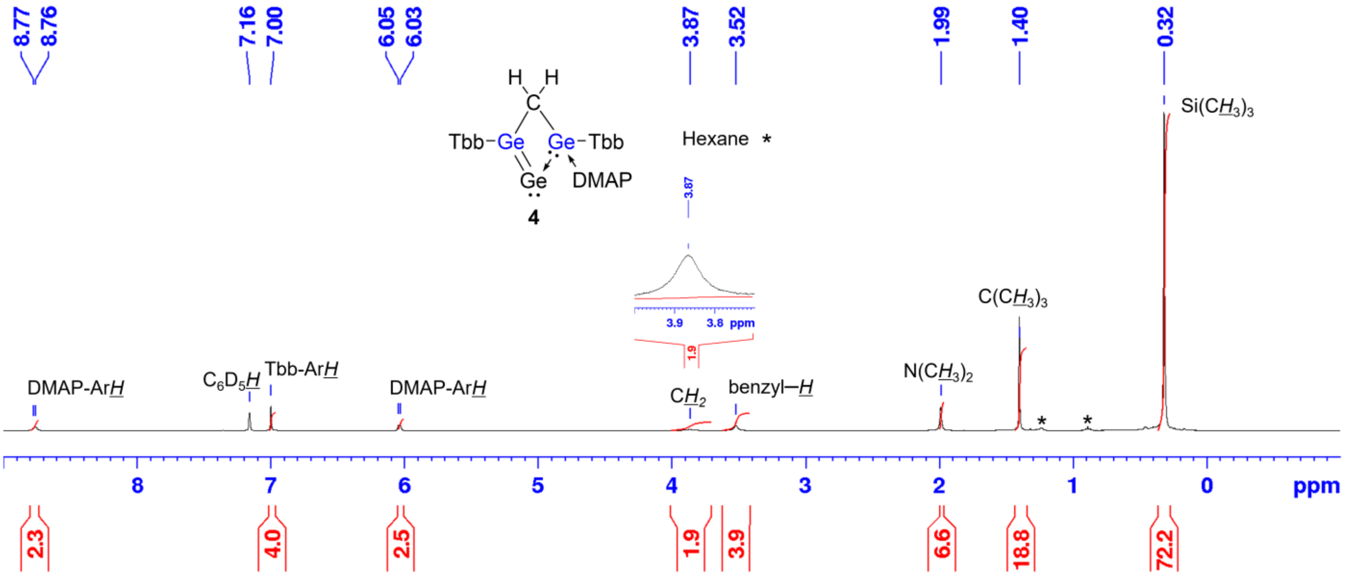

Figure S7. ^{1}H NMR spectrum of **4** (400 MHz, 298 K, C_6D_6).

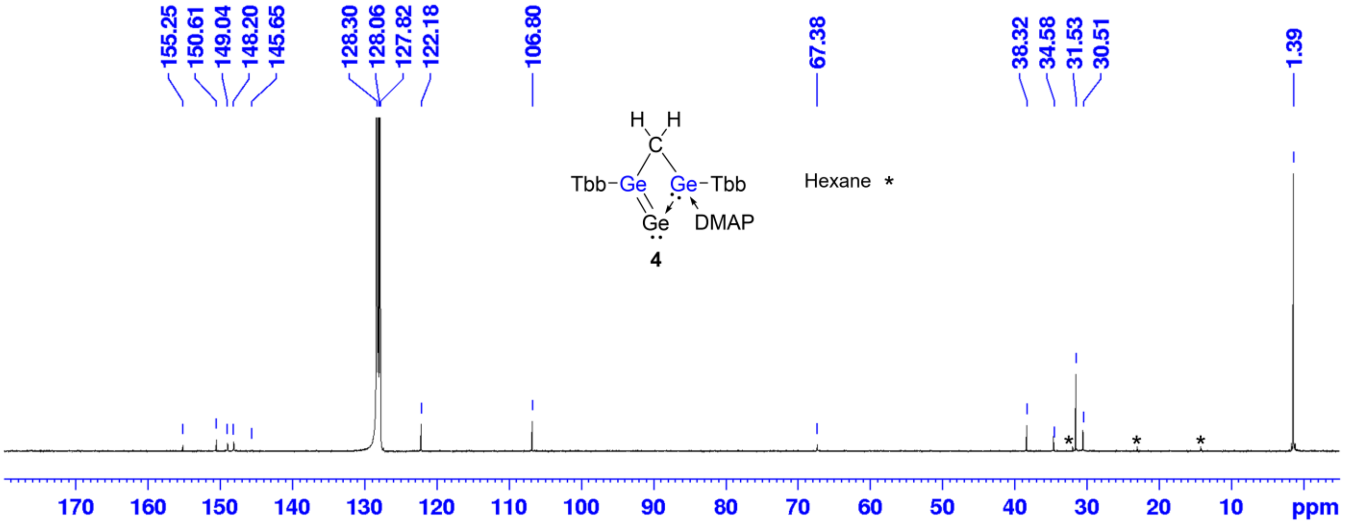

Figure S8. $^{13}C\{^{1}H\}$ NMR spectrum of **4** (101 MHz, 298 K, C_6D_6).

UV/vis Spectra

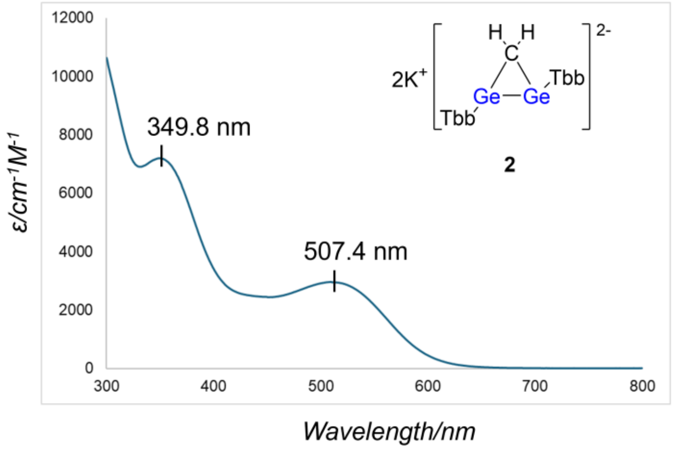

Figure S9. The observed UV/vis spectrum of **2** in benzene.

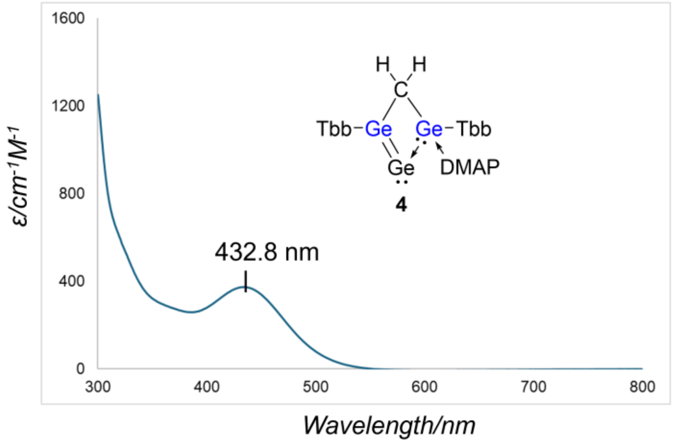

Figure S10. The observed UV/vis spectrum of **4** in benzene.

X-Ray Diffraction Studies

All single crystals were obtained by the slow evaporation technique from benzene. The intensity data were collected 90 K on Bruker D8 VENTURE system (PHOTONIII 14 with IμS Diamond) using Mo Kα radiation (λ = 0.71073 Å). The intensity data were corrected for Lorentz and polarization effects and for absorption (multi-scan). The structures were solved by SHELXT-2018/2[2] and refined by least-squares calculations on F2 for all reflections (SHELXL-2019/3).[3] All non-hydrogen atoms were refined anisotropically. All calculations were performed by using Yadokari-XG[4] and Olex2 1.5.[5] The crystallographic data were summarized in Tables S1. In the CheckCIF report for compound **4**, level B alerts related to the Hirshfeld test for the Ge–Ge bonds were noted. While these may be attributable to an unresolved disorder, the residual electron density and structural parameters suggest that the observed anomaly reflects the genuine thermal motion of the atoms within the crystal.

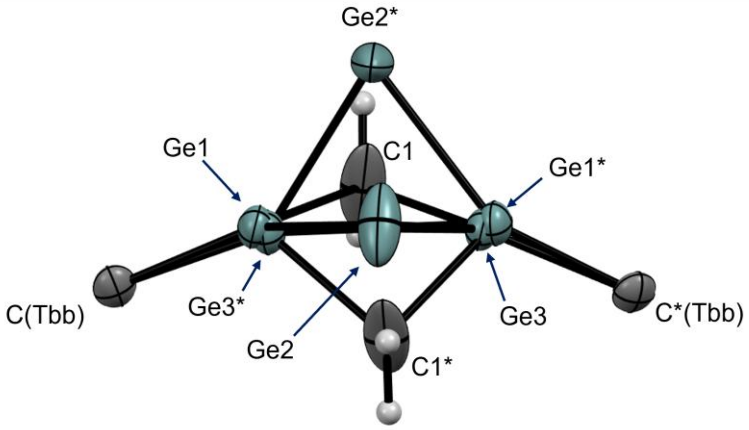

Figure S11. Pseudo-symmetric disorder on the [Ge_3C] ring core of **3**.

Table S1. Crystallographic data for **2**, **3**, and **4**.

	**2**·3C_6H_6	**3**	**4**·C_6H_6
Empirical formula	$C_{67}H_{118}Ge_2K_2Si_8$	$C_{49}H_{100}Ge_3Si_8$	$C_{62}H_{116}Ge_3N_2Si_8$
Formula weight	1371.71	1131.77	1332.05
Temperature (K)	90(2)	90(2)	90(2)
Crystal color	red	red	yellow
Crystal dimensions (mm)	0.20 x 0.10 x 0.040	0.163 x 0.087 x 0.032	0.189 x 0.165 x 0.043
Crystal system	monoclinic	monoclinic	triclinic
Space group	$P2_1/c$ (#14)	$C2/c$ (#15)	P-1 (#2)
a (Å)	22.649(4)	30.202(3)	12.6987(5)
b (Å)	13.124(2)	9.5466(8)	16.2715(6)
c (Å)	26.722(4)	23.1505(17)	19.6919(7)
α (°)	90	90	93.615(1)
β (°)	91.740(6)	103.920(2)	97.189(1)
γ (°)	90	90	109.037(1)
V (Å^3)	7939(2)	6478.8(9)	3792.6(2)
Z	4	4	2
D_{calc} (g·cm^{-3})	1.148	1.160	1.166
μ (mm^{-1})	1.017	1.559	1.342
θ range (°)	1.938 to 27.533	2.001 to 27.524	2.498 to 27.390
Reflections collected	195929	50202	118938
R_{int}	0.1237	0.0909	0.0420
Completeness to θ	99.6	99.5	99.7
No. of restraints	0	0	0
No. of parameters	750	298	696
Goodness of fit	1.027	1.040	1.046
R_1 [I>2σ(I)]	0.0406	0.0567	0.0306
wR_2 [I>2σ(I)]	0.0849	0.1359	0.0764
R_1 (all data)	0.0655	0.0853	0.0399
wR_2 (all data)	0.0959	0.1559	0.0818
Largest diff. peak (e·Å^3)	0.58	0.92	0.69
Largest diff. hole (e·Å^3)	−0.53	−0.87	−0.42
CCDC deposition number	2485175	2485173	2485174

Theoretical Calculations

DFT calculations were performed using the Gaussian 16 (Rev. B. 01) program package.[6] Structural optimizations for **2, 3** and **4** were performed at B3LYP-D3/6–311G(2df,2p) level of theory (D3BJ keyword was used for dispersion correction). The frequency calculations were carried out for each optimized structure to confirm the absence of any imaginary frequencies. Natural bonding orbital (NBO) analysis of **3** and **4** was performed using NBO 7.0 program[7] and calculated at B3LYP-D3/6-311G(2df,2p) level of theory.

Comparison between the experimentally observed and theoretically calculated structures of 3 and 4

Table S2. Selected bond lengths (Å) and angles (°) together with the corresponding theoretical values of **2**.

	Ge1−Ge2	Ge1−C1	Ge2−C1	Ge1−Ge2−C1	Ge1−C1−Ge2	Ge2−Ge1−C1
**2** (obsd.)	2.5818(5)	2.0481(2)	2.0489(2)	50.95(7)	78.13(9)	50.92(7)
2 *(calcd.)*	*2.598*	*2.067*	*2.067*	*51.07*	*77.86*	*51.07*

Table S3. Selected bond lengths (Å) and angles (°) together with the corresponding theoretical values of **3**.

	Ge1−Ge2	Ge2−Ge3	Ge1−Ge3	Ge1−C1	Ge3−C1	Ge1−Ge2−Ge3	Ge1−C1−Ge3
**3** (obsd.)	2.3938(1)	2.3673(1)	2.6718(1)	1.990(11)	1.989(12)	68.27(9)	84.4(4)
3 *(calcd.)*	*2.402*	*2.427*	*2.681*	*2.004*	*1.998*	*67.45*	*84.11*

Table S4. Selected bond lengths (Å) and angles (°) together with the corresponding theoretical values of **4**.

	Ge1−Ge2	Ge2−Ge3	Ge1−Ge3	Ge1−C1	Ge3−C1	Ge3−N	Ge1−Ge2−Ge3	Ge1−C1−Ge3
**4** (obsd.)	2.3376(4)	2.4915(4)	2.8008(3)	1.9980(19)	1.9923(18)	2.0586(16)	70.82(12)	89.16(8)
4 *(calcd.)*	*2.344*	*2.513*	*2.789*	*2.008*	*1.996*	*2.105*	*70.00*	*88.30*

Molecular orbitals of 2, 3 and 4

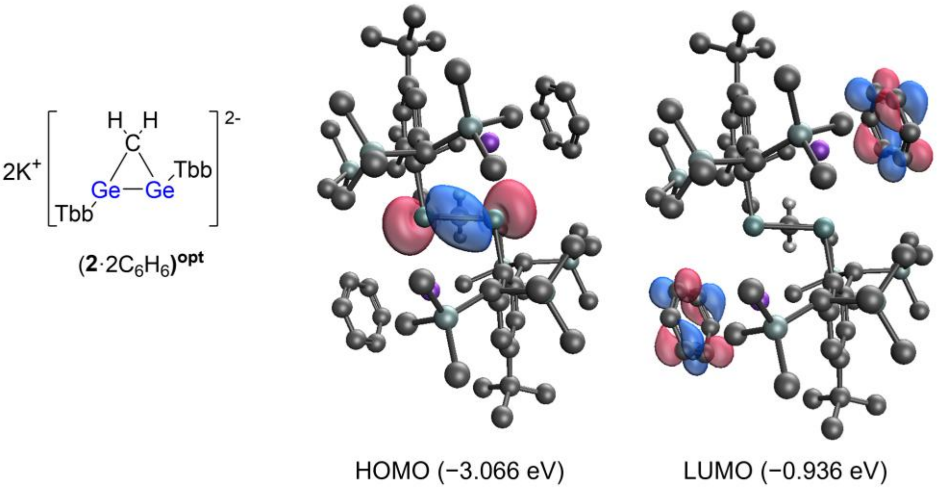

Figure S12. Molecular orbitals of ($\mathbf{2}\cdot 2C_6H_6)^{opt}$ (Isovalue: 0.050).

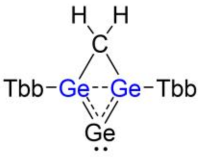

$\mathbf{3}^{\mathrm{opt}}$

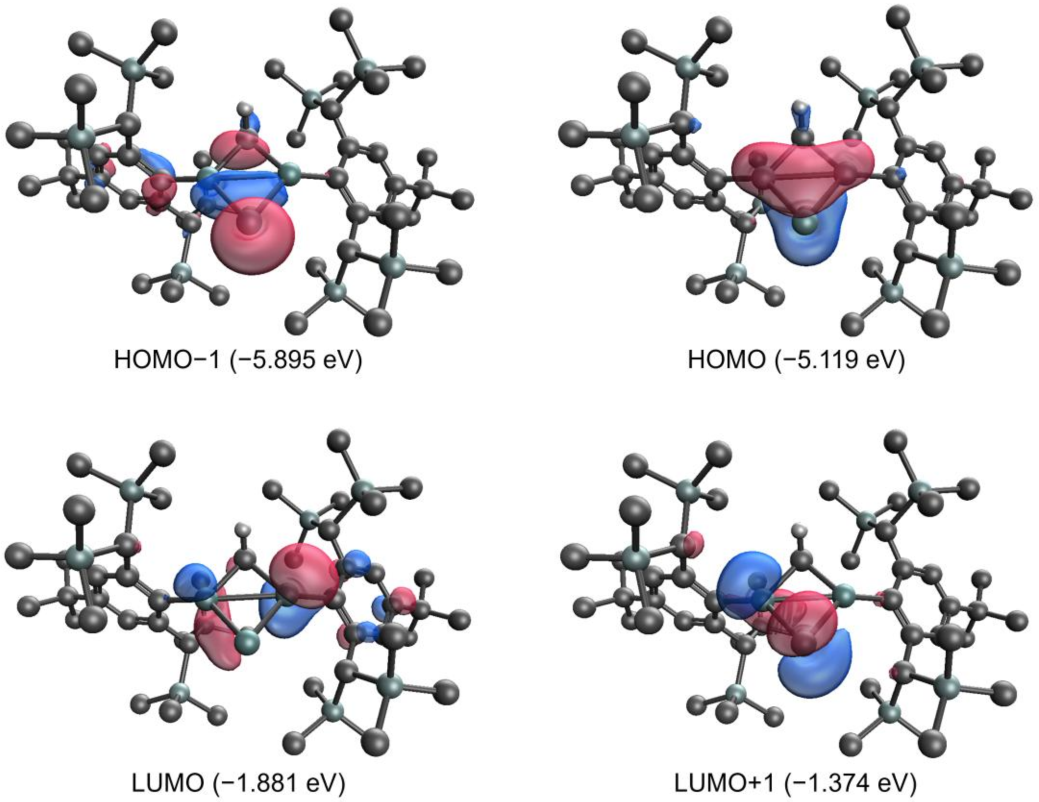

Figure S13. Molecular orbitals of $\mathbf{3}^{\mathrm{opt}}$ (Isovalue: 0.050).

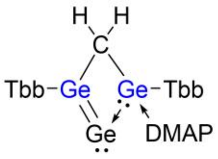

$\mathbf{4}^{\mathrm{opt}}$

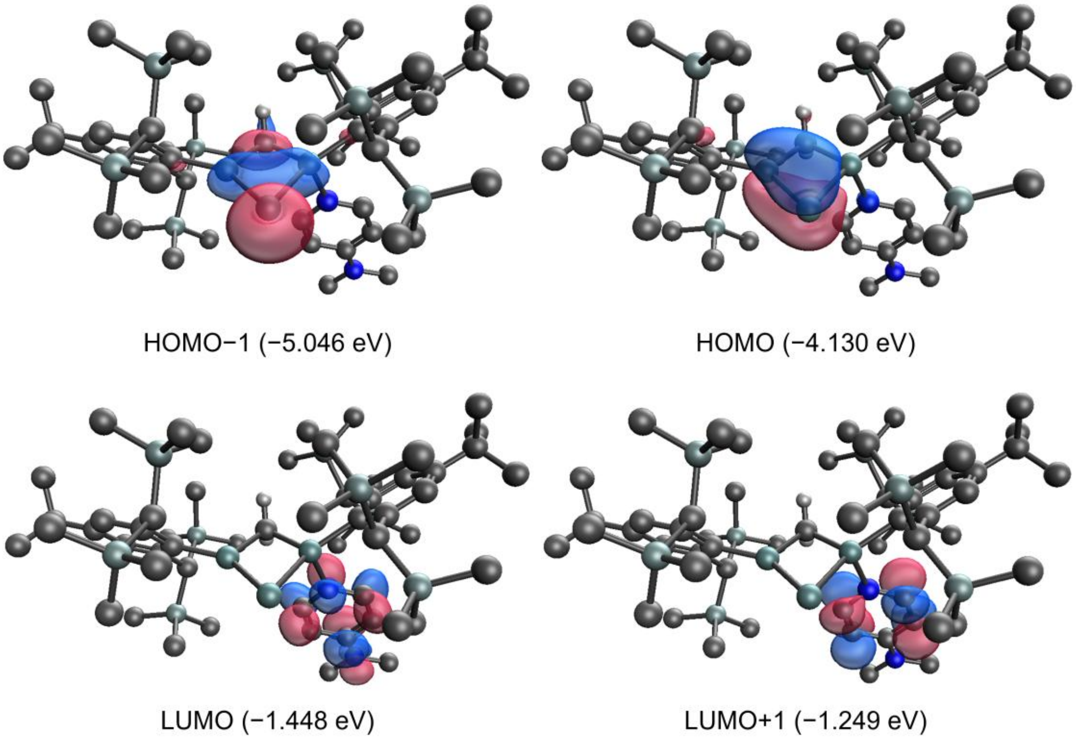

Figure S14. Molecular orbitals of $\mathbf{4}^{\mathrm{opt}}$ (Isovalue: 0.050).

References

[1] D. Uchida, M. Yukimoto, N. Tokitoh, M. Yamauchi, H. Yamada, Y. Mizuhata, *Angew. Chem. Int. Ed.,* **2025**, *64*, e202508927.

[2] G. Sheldrick, *Acta Cryst. A*, **2015**, *71*, 3.

[3] G. Sheldrick, *Acta Cryst. C*, **2015**, *71*, 3.

[4] C. Kabuto, S. Akine, E. Kwon, *J. Cryst. Soc. Jpn.,* **2009**, *51*, 218.

[5] O. V. Dolomanov, L. J. Bourhis, R. J. Gildea, J. A. K. Howard, H. Puschmann, H. *J. Appl. Cryst*., **2009**, *42*, 339.

[6] Gaussian 16, Revision B.01, M. J. Frisch, G. W. Trucks, H. B. Schlegel, G. E. Scuseria, M. A. Robb, J. R. Cheeseman, G. Scalmani, V. Barone, G. A. Petersson, H. Nakatsuji, X. Li, M. Caricato, A. Marenich, J. Bloino, B. G. Janesko, R. Gomperts, B. Mennucci, H. P. Hratchian, J. V. Ortiz, A. F. Izmaylov, J. L. Sonnenberg, D. Williams-Young, F. Ding, F. Lipparini, F. Egidi, J. Goings, B. Peng, A. Petrone, T. Henderson, D. Ranasinghe, V. G. Zakrzewski, J. Gao, N. Rega, G. Zheng, W. Liang, M. Hada, M. Ehara, K. Toyota, R. Fukuda, J. Hasegawa, M. Ishida, T. Nakajima, Y. Honda, O. Kitao, H. Nakai, T. Vreven, K. Throssell, J. A. Montgomery Jr, J. E. Peralta, F. Ogliaro, M. Bearpark, J. J. Heyd, E. Brothers, K. N. Kudin, V. N. Staroverov, T. Keith, R. Kobayashi, J. Normand, K. Raghavachari, A. Rendell, J. C. Burant, S. S. Iyengar, J. Tomasi, M. Cossi, J. M. Millam, M. Klene, C. Adamo, R. Cammi, J. W. Ochterski, R. L. Martin, K. Morokuma, O. Farkas, J. B. Foresman, D. J. Fox, Gaussian, Inc., Wallingford CT, 2016.

[7] E. D. Glendening, J, K. Badenhoop, A. E. Reed, J. E. Carpenter, J. A. Bohmann, C. M. Morales, P. Karafiloglou, C. R. Landis, F. Weinhold, NBO 7.0 (2018).

Author Contributions

Daichi Uchida: Investigation, Writing - Original Draft, Writing - Review & Editing
Hiroko Yamada: Writing - Review & Editing
Yoshiyuki Mizuhata: Conceptualization, Investigation, Writing - Original Draft, Writing - Review & Editing
